# Vaginal artery pseudoaneurysm after a hysterectomy: A case report and review of the literature

**DOI:** 10.1002/ccr3.9006

**Published:** 2024-06-27

**Authors:** Fariba Zabihi, Somayeh Moeindarbari, Farzaneh Khoroushi, Mohammad Mishan, Khosrow Ravankhah Moghaddam

**Affiliations:** ^1^ General Surgery Department Mashhad University of Medical Sciences Mashhad Iran; ^2^ Department of Obstetrics and Gynecology, Neonatal and Maternal Research Center Mashhad University of Medical Sciences Mashhad Iran; ^3^ Department of Radiology, Faculty of Medicine Mashhad University of Medical Sciences Mashhad Iran; ^4^ Faculty of Medicine Abadan University of Medical Sciences Abadan Iran; ^5^ Surgical Oncology Research Center Mashhad University of Medical Sciences Mashhad Iran

**Keywords:** aneurysm, case reports, hysterectomy, vagina

## Abstract

**Key clinical message:**

This was the first report of a pseudoaneurysm in a vaginal artery after hysterectomy, unlike other published studies that were of pseudoaneurysms in uterine or vaginal arteries after delivery.

**Abstract:**

A 51‐year‐old woman presented with massive vaginal bleeding 7 days after a hysterectomy, which caused hemoglobin to drop. The patient was suspicious of having a vaginal artery pseudoaneurysm according to the sonography. Her bleeding was stopped after the ligation of her left internal iliac artery.

## 1 INTRODUCTION

Hysterectomy is believed to be the second most frequent type of gynecologic surgery after cesarean section, all around the world.^1^ Surgery can be conducted through abdominal or vaginal access and can be total or partial. It is mainly indicated in patients with uterine fibroids, endometriosis, genital prolapse, pelvic pain, heavy menstrual bleeding, or gynecological malignancies.^1^, ^2^

Post‐surgery hemorrhage is an infrequent complication of hysterectomy; however, it can be life‐threatening. The incidence of hemorrhage depends on the type of surgery and surgeon expertise, but it constitutes an incidence of 0.2 to 3.1 percent.^3^ This condition may happen regardless of the method of hysterectomy and can happen early or reactionary and delayed or secondary. Timely recognition and prompt intervention to halt the bleeding are essential for a favorable outcome for the patient.^3^, ^4^

One of the rare causes of post‐surgery hemorrhage is the rupture of a pseudoaneurysm of the uterine or vaginal arteries. It is reported that vaginal artery pseudoaneurysm can mainly occur due to uterine dilation and curettage. However, it can infrequently be caused by pelvic surgeries like hysterectomy.^5^ Several reports of postpartum uterine artery or vaginal artery pseudoaneurysms have been published.^5^, ^6^, ^7^ Still, to our best knowledge, no report addressed vaginal artery pseudoaneurysm after hysterectomy. Here, we report a case of vaginal artery pseudoaneurysm following elective hysterectomy in a 51‐year‐old woman.

## 2 CASE PRESENTATION

### 2.1 Case history/examination

A 51‐year‐old Iranian female para four (including three live deliveries and one abortion) was admitted to the emergency room with severe vaginal bleeding. She had a hysterectomy 1 week before her admission due to multiple fibromas and abnormal vaginal bleeding. Moreover, her history was positive for a peptic ulcer and hypertension from 8 years ago. Also, she had a colpography 3 years ago. In terms of drug history, she had a prescription for 25 mg of losartan QD and 80 mg of aspirin QD. Family and social history had no notable findings.

The patient's vital sign assessment showed a blood pressure of 110/80 mmHg, a pulse rate of 80 beats per second, a respiratory rate of 18 per minute, and a temperature of 37°C. In the case of physical examination, abdominal appearance showed healing midline sutures without any signs of infection. Moreover, the abdomen was soft and non‐tender in the physical examination. The perineal examination was normal.

## 3 METHODS (DIFFERENTIAL DIAGNOSIS, INVESTIGATIONS, AND TREATMENT)

The patient had a laboratory examination that showed a white blood cell count of 19,300 per μL with a polymorphonuclear of 94.0%, a red blood cell count of 3.1 million per μL, and hemoglobin of 8.3 g/dL with a hematocrit of 26.9%. Furthermore, blood sugar was 165 mg/dL, urea of 18 mg/dL, and creatinine of 1.1 mg/dL. Furthermore, coagulation tests revealed a prothrombin time (PT) of 13 s, partial thromboplastin (PTT) of 33 s, and an international normalized ratio (INR) of 1.12.

The patient underwent sonography that showed suspicious blood flow for pseudoaneurysm in the left part of the vaginal cuff. There were no pathologic findings in the adnexa. Also, no signs of free fluid or hematoma in abdominopelvic cavity were present on sonography.

The patient underwent laparotomy from the previous incision site in order to find the site of hemorrhage. The inspection of abdominopelvic cavity showed no site of active bleeding; however, even after repairment of the vaginal cuff, there was an amount of vaginal bleeding. After the further inspection of vaginal cuff, a pseudoaneurysm site was detected in the left posterior vaginal artery, which was resected for further pathology assessment.

## 4 CONCLUSION AND RESULTS (OUTCOME AND FOLLOW UP)

**FIGURE 1:**
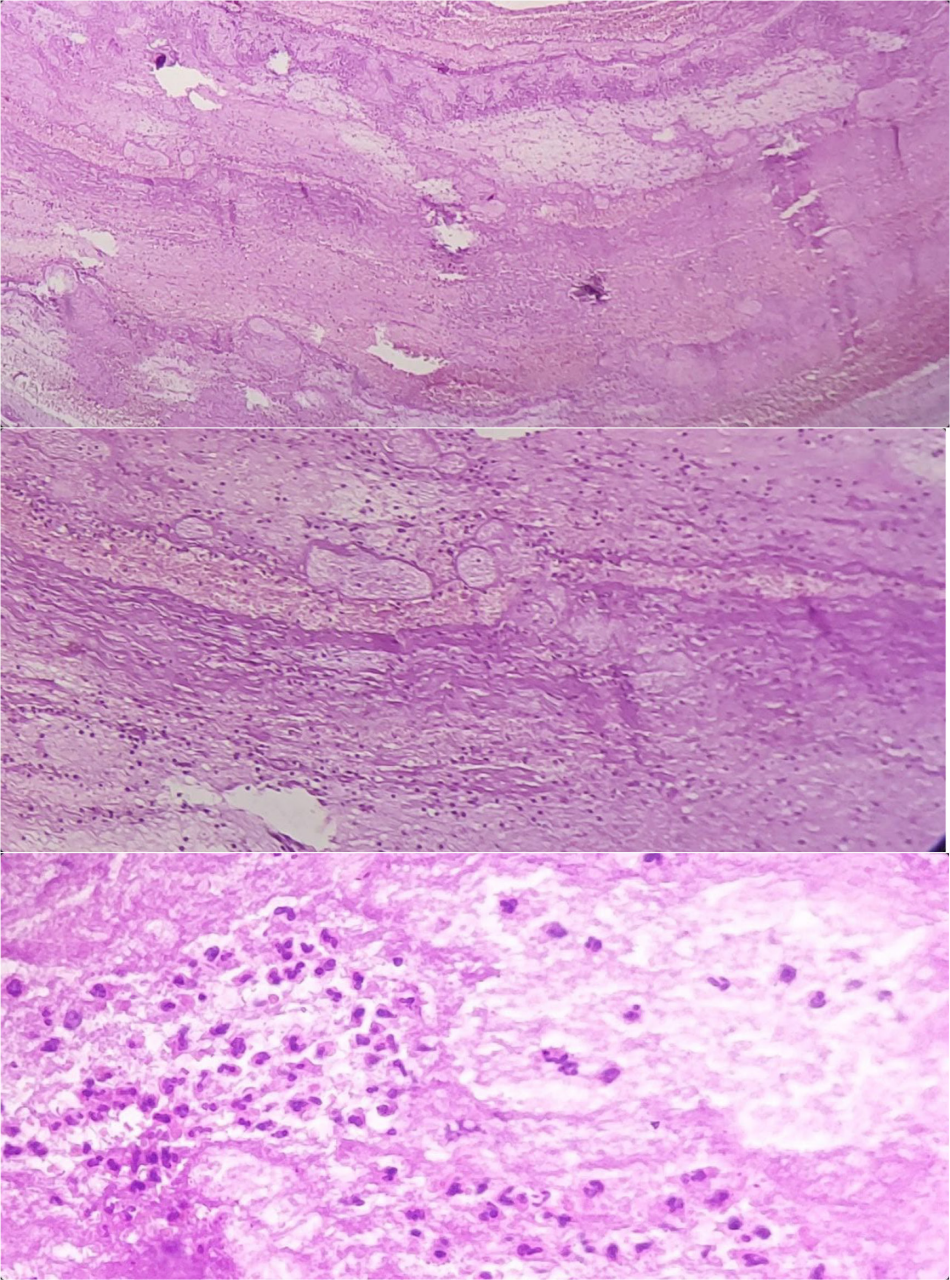
Pathology views of the pseudoaneurysm showed fibrin deposition and leukocyte infilterate.

The hemorrhage stopped after ligation of the left internal iliac artery. Pathology assessment confirmed a pseudoaneurysm of the left vaginal artery. Figure 1 shows the pathology slide of the assessed vaginal pseudoaneurysm.

## 5 DISCUSSION

The uterus and upper part of the vagina are supplied with two main arteries named as uterine artery and vaginal artery, respectively, which both originate from the internal iliac artery. There are several reports in case of iatrogenic vascular injury during uterine curettage or surgical trauma that are classified as arteriovenous malformations (AVMs), arteriovenous fistulas, direct vessel ruptures, and pseudoaneurysms.^8^, ^9^ An arterial pseudoaneurysm is the formation of a periarterial bloody cyst that communicates with the arterial lumen through a narrow neck. It seems that pseudoaneurysms are more infrequent than other injuries. The arterial pseudoaneurysm differs from true aneurysm, because its walls are formed of only one or two layers with respect to the three layers of arterial walls and the presence of a narrow neck.^10^, ^11^

Youssef et al.^10^ reported two cases of intrauterine arterial pseudoaneurysm. The first case was a postpartum hemorrhage after cesarean section in a 26‐year‐old woman. She referred 10 days after delivery with massive vaginal bleeding and her hemoglobin dropped to 3 g/dL 48 h after the admission. However, the patient's bleeding ceased even without any surgical intervention. The other case was a 30‐year‐old female, who was admitted with heavy vaginal bleeding 10 days after a miscarriage and subsequent uterine curettage. Her bleeding was also managed with a hysterectomy.^10^

Besides the iatrogenic rupture of uterine and vaginal arteries, there are reports of the pseudoaneurysm of these two arteries after vaginal delivery that constitute a rare cause of postpartum hemorrhage. A retrospective review of 588 pelvic angiography, showed only 20 cases of pseudoaneurysm, and among them, three cases demonstrated left vaginal artery pseudoaneurysm and most of the cases had uterine artery pseudoaneurysm.^12^

Another study that used a 7‐year hospital record reported seven cases of severe postpartum hemorrhage from ruptured pseudoaneurysms. Four cases had no prior history of intervention and were spontaneous; one case was after surgical abortion; one case was after vaginal delivery with forceps; and one was after a cesarean section. Still, among these, two had uterine artery pseudoaneurysm and four had vaginal artery pseudoaneurysm. All the women in this case study were at child bearing age ranging between 28 and 39 years old.^13^

Even bizarre presentation of the uterine artery pseudoaneurysm rupture is reported. Anwer et al.^14^ proposed a 25‐year‐old female, who presented with shock after a 2‐month period of massive hematuria accompanied a delivery history. They suspected the diagnosis using CT angiography and the patient was treated with angioembolization.

However, our case differed from those that were found in the literature. Our patient was a woman in her 50's, who had a secondary vaginal hemorrhage after hysterectomy but not in a postpartum manner. Moreover, the pseudoaneurysm was in the vaginal artery, which seems to be less frequent than uterine artery pseudoaneurysm.

The diagnosis of this condition is based on suspicion, as it is not a common pathologic finding. Angiography can be very helpful in the diagnosis and treatment of patients. However, it is not widely accessible in all medical centers. The important point is to avoid hard transvaginal examination. Sonography can provide a clue to the diagnosis and further surgical approach can find the site of aneurysm.^11^, ^15^, ^16^

Furthermore, angiographic embolization is widely used in studies as a treatment. This is usually conducted in a bilateral manner in order to avoid the chance of hemorrhage due to possible collateral feeding arteries. But the method is not widely available. Therefore, other treatment approaches like hysterectomy and surgical ligation of internal iliac arteries have also been conducted.^17^ In our case, after hysterectomy, and due to the absence of an angiographic method, surgical ligation of the left internal iliac artery was conducted.

## 6 CONCLUSION

In case of abnormal vaginal bleeding, many differential diagnoses are proposed; however, vaginal artery pseudoaneurysm is very rare among them. This pathology is usually found in postpartum women and after dilation and curettage. We also added that vaginal artery pseudoaneurysm after hysterectomy can happen. The condition has nonspecific manifestations like vaginal bleeding and can be detected after ruling out other possible causes. Sonography can guide the surgeon to the diagnosis and angioembolization or ligation of the internal iliac artery is reported to be the cure for this disorder.

## AUTHOR CONTRIBUTIONS

**Fariba Zabihi:** Conceptualization; data curation; methodology; writing – original draft. **Somayeh Moeindarbari:** Conceptualization; investigation; methodology. **Farzaneh Khoroushi:** Visualization; writing – original draft. **Mohammad Mishan:** Methodology; writing – original draft. **Khosrow Ravankhah Moghaddam:** Supervision; writing – review and editing.

## FUNDING INFORMATIONS

The patient had a laboratory examination that showed a white blood cell count of 19,300 per uL with a polymorphonuclear of 94.0%, a red blood cell count of 3.1 million per pL, and hemoglobin of 8.3 g/dL with a hematocrit of 26.9%. Furthermore, blood sugar was 165 mg/dL, urea of 18 mg/dL, and creatinine of 1.1 mg/dL. Furthermore, coagulation tests revealed a prothrombin time (PT) of 13s, partial thromboplastin (PTT) of 33 s, and an international normalized ratio (INR) of 1.12.The patient underwent sonography that showed suspicious blood flow for pseudoaneurysm in the left part of the vaginal cuff.

## CONFLICT OF INTEREST STATEMENT

The authors declare that there are no conflicts of interest.

## ETHICS STATEMENT

This study was approved by the Ethics Committee of Mashhad University of Medical Sciences, Mashhad, Iran. All tests were carried out in compliance with the institution's specified rules and regulations. Furthermore, the patient's written informed consent was acquired for the publishing of this case report.

## CONSENT

Written informed consent was obtained from the patient to publish this report in accordance with the journal's patient consent policy.

## Data Availability

The data are also available on request.
